# Study protocol subacromial impingement syndrome: the identification of pathophysiologic mechanisms (SISTIM)

**DOI:** 10.1186/1471-2474-12-282

**Published:** 2011-12-14

**Authors:** Pieter Bas de Witte, Jochem Nagels, Ewoud RA van Arkel, Cornelis PJ Visser, Rob GHH Nelissen, Jurriaan H de Groot

**Affiliations:** 1 Department of Orthopaedics, Leiden University Medical Centre (LUMC), Postzone J11R, Postbus 9600, 2300 RC Leiden, The Netherlands; 2 Department of Orthopaedics, Medical Centre Haaglanden (MCH), Postbus 432, 2501 CK Den Haag, The Netherlands; 3 Department of Orthopaedics, Rijnland Hospital, Simon Smitweg 1, 2353 GA Leiderdorp, The Netherlands; 4 Laboratory for Kinematics and Neuromechanics, Departments of Rehabilitation and Orthopaedics, Leiden University Medical Centre, Postzone B0-Q, Postbus 9600, 2300 RC Leiden, The Netherlands

## Abstract

**Background:**

The Subacromial Impingement Syndrome (SIS) is the most common diagnosed disorder of the shoulder in primary health care, but its aetiology is unclear. Conservative treatment regimes focus at reduction of subacromial inflammatory reactions or pathologic scapulohumeral motion patterns (*intrinsic *aetiology). Long-lasting symptoms are often treated with surgery, which is focused at enlarging the subacromial space by resection of the anterior part of the acromion (based on *extrinsic *aetiology). Despite that acromionplasty is in the top-10 of orthopaedic surgical procedures, there is no consensus on its indications and reported results are variable (successful in 48-90%). We hypothesize that the aetiology of SIS, i.e. an increase in subacromial pressure or decrease of subacromial space, is multi-factorial. SIS can be the consequence of pathologic scapulohumeral motion patterns leading to humerus cranialisation, anatomical variations of the scapula and the humerus (e.g. hooked acromion), a subacromial inflammatory reaction (e.g. due to overuse or micro-trauma), or adjoining pathology (e.g. osteoarthritis in the acromion-clavicular-joint with subacromial osteophytes).

We believe patients should be treated according to their predominant etiological mechanism(s). Therefore, the objective of our study is to identify and discriminate etiological mechanisms occurring in SIS patients, in order to develop tailored diagnostic and therapeutic strategies.

**Methods:**

In this cross-sectional descriptive study, applied clinical and experimental methods to identify intrinsic and extrinsic etiologic mechanisms comprise: MRI-arthrography (eligibility criteria, cuff status, 3D-segmented bony contours); 3D-motion tracking (scapulohumeral rhythm, arm range of motion, dynamic subacromial volume assessment by combining the 3D bony contours and 3D-kinematics); EMG (adductor co-activation) and dynamometry instrumented shoulder radiographs during arm tasks (force and muscle activation controlled acromiohumeral translation assessments); Clinical phenotyping (Constant Score, DASH, WORC, and SF-36 scores).

**Discussion:**

By relating anatomic properties, kinematics and muscle dynamics to subacromial volume, we expect to identify one or more predominant pathophysiological mechanisms in every SIS patient. These differences in underlying mechanisms are a reflection of the variations in symptoms, clinical scores and outcomes reported in literature. More insight in these mechanisms is necessary in order to optimize future diagnostic and treatment strategies for patients with SIS symptoms.

**Trial registration:**

Dutch Trial Registry (Nederlands Trial Register) NTR2283.

## Background

### Introduction

The Subacromial Impingement Syndrome (SIS) can be defined as symptomatic irritation of the rotator cuff and subacromial bursa in the limited subacromial space. Clinical characteristics are pain with arm abduction (painful arc), decreased active range of motion (RoM) and loss of arm force and function [1-5]. It is the most frequently diagnosed shoulder disorder in primary health care, accounting for 44-65% of all shoulder complaints [3,6]. Symptoms can persist for months or years and the majority of patients are between 40 and 50 years old. Consequently, SIS has a significant socioeconomic impact [7].

Despite its reported prevalence, the diagnostic criteria and aetiology of SIS are debatable. Two main etiologic theories have been described. Neer's widely accepted impingement theory focuses on the *extrinsic *mechanism: symptoms result from compressive forces on the rotator cuff, caused by biomechanical or structural anatomic (bony) abnormalities [8,9]. The mechanisms leading to this assumed compression remain unclear. Scapula dyskinesia, causing relative cranial translation of the humerus, has been reported [6,10-13]. Other studies describe a correlation between SIS and acromial shape (hooked acromion, Bigliani classification [14] type II or III) [4,15-18]. Presumably, this hooked acromion is a pre-existing anatomic variation or a traction spur on the coracoacromial ligament caused by repetitive cranially directed translations of the humerus or by tendinopathy. Others conclude there is no relation between acromial shape and SIS, or underline the difficulties in using acromial shape as an assessment tool [16,19,20]. The majority of partial rotator cuff tears, commonly referred to as a consequence or entity of SIS, are often either intratendinous or at the articular side of the rotator cuff and not at the bursal side where they would be expected if the rotator cuff 'impinges' against a hooked acromion [21]. Despite these unclarities, the extrinsic mechanism forms the rationale for one of the most frequently performed orthopaedic surgical procedures: acromionplasty. The second theory is based on a degenerative *intrinsic *mechanism: SIS can be caused by ischemia at the watershed zone of the supraspinatus tendon. This is enhanced by micro traumata or overuse, tensile overload on degenerating rotator cuff tendons, a subacromial inflammatory reaction, or insufficient cuff function leading to an imbalance between glenohumeral mobility and joint stability, with consequent glenohumeral destabilization or altered arm-shoulder kinematics [22-29]. Thirdly, SIS can be the consequence of adjoining pathologies or joint hyperlaxity. Furthermore, less classic forms of shoulder impingement, e.g. internal impingement and coracoid impingement have been described.

Treatment of SIS generally starts with conservative methods, including arm rest or physical therapy, non-steroidal anti-inflammatory drugs (NSAIDs) and subacromial corticosteroids injections. Conservative therapy is successful in 42% (Bigliani type III) to 91% (Bigliani type I) [30,31]. When conservative treatments fail, the classic surgical treatment of primary SIS is an acromionplasty as described by Neer [8,9]. Variable and often mediocre results of this frequently applied procedure have been reported, with success rates ranging from 48 to 90% [32-36]. However, acromionplasty doesn't affect continuing degeneration of the rotator cuff [37], and subacromial spur recurrence has been reported following acromionplasty [21,38,39]. Henkus et al. reported comparable results for acromionplasty and bursectomy in patients with SIS [40]. This is in concordance with other studies that also report clinical improvements in SIS-patients without changing the coracoacromial shape [31,40-44].

Although SIS has been typically assumed to be the result of rotator cuff injury, the subacromial space is a complex anatomical environment, containing several structures that can be a source of pain. Even several pathologies that have a similar patients' history, pain patterns and findings with physical examination, can be (mistakenly) diagnosed as SIS [45]. In a recent study at our institution, 14 of 80 patients (17.5%) clinically diagnosed with SIS, had to be excluded following MRI arthrography because of alternative shoulder pathology [40].

Concluding, the ongoing debate on the aetiology of SIS, its varying clinical presentations, the diagnostic difficulties and the highly variable treatment outcomes of SIS suggest there might be multiple pathophysiologic mechanisms leading to complaints clinically diagnosed as SIS that need specific approaches in clinical practice.

### Hypothesis

The extrinsic pathophysiologic mechanism is only valid for a subgroup of SIS patients, and consequently acromionplasty is the wrong treatment for at least a part of the patients suffering from SIS symptoms. The complaints observed in SIS are presumably a compilation of symptoms that originate from different shoulder pathologies and etiologic mechanisms. It is our challenge to discriminate these intrinsic and/or extrinsic underlying aetiologies.

**Figure 1:**
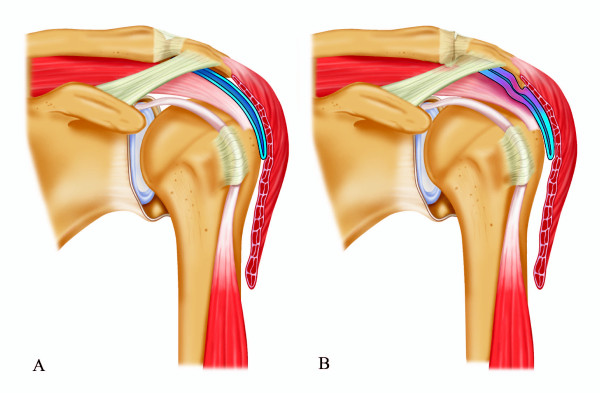
**A. Schematic anatomy of a healthy glenohumeral joint and subacromial space. B. Schematic anatomy of a shoulder joint with the presence of several etiologic mechanisms for Subacromial Impingement Syndrome.** In theory, impingement ("a disbalance between acromial space and the space needed for subacromial structures") can be caused by 1) A dynamically reduced subacromial space due to a pathologic pattern of arm-shoulder movements (e.g. scapular dyskinesia), resulting in relative cranialisation of the humerus with respect to the scapula/acromion, or 2) A more statically reduced subacromial space, due to 2a) structural anatomic variations (e.g. a hooked acromion), eventually in combination with altered arm-scapula motion patterns; 2b) A subacromial inflammatory reaction (e.g. caused by micro-trauma or overuse) causing subacromial oedema, fibrosis and tendinosis; 2c) Encroachment of subacromial tissues by an adjoining pathology or structures other than the acromion (e.g. acromioclavicular (AC)-joint osteoarthritis and subacromial osteophytes, calcifying tendinitis, and coracoid impingement).

We developed a theoretical framework for the aetiology of impingement ("a disbalance between subacromial volume and the space needed for subacromial structures", i.e. increased subacromial pressure) based on 4 distinct proposed mechanisms (Figure 1):

1) A dynamically reduced subacromial space due to a pathologic pattern of arm-shoulder movements (e.g. scapular dyskinesia), resulting in relative cranialisation of the humerus with respect to the scapula/acromion.

2) A more statically reduced subacromial space, due to:

a. structural anatomic variations (e.g. a hooked acromion), eventually in combination with altered arm-scapula motion patterns;

b. a subacromial inflammatory reaction (e.g. caused by micro-trauma or overuse) causing subacromial oedema, fibrosis and tendinosis;

c. Encroachment of subacromial tissues by an adjoining pathology or structures other than the acromion (e.g. acromioclavicular (AC)-joint osteoarthritis and subacromial osteophytes, calcifying tendinitis, and coracoid impingement).

In this study, factors associated with these SIS mechanisms will be analyzed in patients clinically diagnosed with SIS. As a result, SIS patients will be categorised in "dynamic" and "static" etiologic subgroups, each requiring tailored diagnostics and treatment strategies.

Because subacromial impingement syndrome is a clinical diagnosis, possible other causes of shoulder pain and SIS symptoms (e.g. early stage frozen shoulder, calcifying tendinitis, slap lesions, rotator cuff tears, etc.) are identified and, if eligible, analyzed separately in distinct research projects (trial registry numbers: NTR1545 and NTR2282).

### Study goals

#### Primary goal

Identification and classification of distinct pathophysiological mechanisms for symptoms clinically diagnosed as SIS into identifiable subgroups of patients as categorized above, in order to design tailored concept diagnostics and treatment flowcharts from experimental concepts.

#### Secondary goals

A set of experimental and diagnostic tools is combined to identify structural and biomechanical etiological factors in SIS patients, which will be related to clinical and functional status:

A. Presence and severity of pathologies in the subacromial space with MRI, e.g. (partial) cuff-ruptures, tendinosis, fibrosis or a subacromial inflammatory reaction, and assessment of cuff degradation status.

B. Acromial shape classification and 3D shape parameters of the humerus, scapula and subacromial space volume, using conventional radiographs and segmented MRI-arthrographies.

C. Quantification of cranialisation of the humerus with respect to the scapula at rest and during active arm abduction and adduction tasks with simultaneously acquired shoulder radiographs and Electromyography (EMG) recordings (see D).

D. Measurements of the activation of arm adductors during arm abduction tasks and assessment of the presence of arm adductor co-activation (Activation Ratio).

E. Analyses of 3D-kinematics (arm range of motion and scapulohumeral rhythm) of the affected SIS shoulder compared to the unaffected shoulder and eventual etiologic SIS subgroups, with the use of 3D motion registration.

F. Changes in reconstructed subacromial volume and acromiohumeral distance during arm abduction by combining the recorded 3D-kinematics with the MRI-segmented 3D bony shapes.

G. The effect of a subacromial infiltration of lidocaïne on arm range of motion, scapulohumeral and -thoracic rhythm with arm abduction, reconstructed subacromial volume and muscle activation patterns, including adductor co-activation.

H. Biomechanical analyses of structural or coordinative muscular imbalance by means of model simulation (Delft Shoulder and Elbow Model) with recorded 3D-kinematics as input.

I. Clinical phenotyping, using validated clinical scores and questionnaires.

J. Identification of alternative diagnoses that may cause complaints clinically diagnosed as SIS, using MRI and radiographs (e.g. acromioclavicular-osteoarthritis and subacromial osteophytes, calcifying tendinitis, SLAP lesion or coracoid impingement).

## Methods

### Study design

In this multicentre observational cohort study, patients clinically diagnosed with subacromial impingement syndrome in either one of 3 participating hospitals (Leiden University Medical Centre (LUMC), Medical Centre Haaglanden (MCH), Rijnland Hospital Leiderdorp) will be included for analyses at the LUMC Laboratory for Kinematics and Neuromechanics.

The Medical Ethics Committee of the university medical centre approved all stages of the study. Written informed consent will be obtained from all patients.

### Study population

#### Selection of participants

Patients will be recruited by 3 orthopaedic surgeons involved in the 3 participating hospitals. Patients will be selected if one or more of the following (limited) usual care criteria are present, next to a positive Neer impingement test (lidocaine) and a positive Hawkins test:

Patients' history:

◦ diffuse unilateral shoulder pain for > 3 months;

◦ pain during activities with abduction, retroflexion and/or internal rotation (e.g. closing the door, putting on jacket, overhead activities);

◦ pain at night or incapable of lying on the shoulder.

Physical examination:

◦ positive Yocum test;

◦ painful arc;

◦ diffuse pain at palpation of the greater tuberosity;

◦ disturbed scapulohumeral rhythm;

◦ no signs of pathologies or symptoms on the controlateral shoulder;

◦ capable of 90 degrees of passive abduction and 90 degrees of external rotation.

After the first and clinical inclusion round, symptoms of eligible patients are further investigated with the use of standard shoulder radiographs (anteroposterior in both external and internal rotation and Y scapular view (scapular outlet view)) and an MRI-arthrography of the shoulder. The MRI-arthrographies are evaluated at the local hospital for clinical purposes and additionally evaluated by one of two participating musculoskeletal radiologists at LUMC for eligibility criteria and assessment of acromion classification and rotator cuff status.

Patients are excluded if one of the following characteristics is found with the visit to the outpatient clinic, standard shoulder radiographs or MRI-arthrography:

◦ Age below 35 years or above 60 years;

◦ Restrictions in passive movements of the glenohumeral joint (adhesive capsulitis);

◦ History of fracture or dislocation of the shoulder, history of surgery around the shoulder;

◦ Co-morbidities on the affected shoulder (including fractures, benign or malignant tumours, labrum abnormalities, Hill Sachs lesion, capsular or ligamentous abnormalities, glenohumeral instability, glenohumeral movement restriction, glenohumeral or symptomatic acromioclavicular osteoarthritis, rheumatic disorder, biceps muscle tendinitis, complete (full thickness) rotator cuff rupture, cervical radiculopathy, PASTA lesion, Pulley lesion, calcifying tendinitis > 3 mm, or neurological deficits);

◦ Symptoms on the controlateral shoulder;

◦ No informed consent.

Patients with either rotator cuff tears or calcifying tendinitis are included, if eligible, in separate research projects.

#### Sample size

A combination of techniques will be applied to classify SIS-patients into pathophysiologic subgroups, most of which are newly developed (section 2.3). The acromiohumeral distance (AH) is a recognized parameter related to rotator cuff disease and based on literature it has rather wide inter-individual variations. Therefore, sample size calculation will be based on this parameter.

In a study of Gruber et al., AH values of 9.4 (SD = 3.4) were observed in subjects without diagnosed cuff pathology. A subacromial space narrower than 6 mm on radiographs is considered pathologic and strongly indicative for supraspinatus tendon rupture [46].

The unpaired *t*-test was used to determine the sample size with a difference of AH of 3.4 mm between groups assumed as clinically relevant, comparing AH during abduction task radiographs in patients where humerus cranialisation plays a key-role compared to AH in other subgroups of SIS patients.

Based on the standardized difference: 3.4 mm/3.4 mm = 1.0, a required power of 80% and a p-value of 0.05 for significance, the Altman's Nomogram resulted in 30 shoulders/patients per group.

In our hypothesis, we defined 4 etiological mechanisms. Based on clinical experience and literature, we assume around 30% type III acromion responsible for complaints of SIS,[16] 20-30% of the SIS symptoms are caused by humerus cranialisation and pathologic motion patterns,[47] 15-20% by subacromial inflammatory processes without subacromial narrowing and 5-10% by other impinging structures than the acromion, leaving around 10-30% for a group in which SIS symptoms seem to be caused by two or more hypothesized etiologic mechanisms. With 30 patients needed in the humerus cranialisation subgroup (i.e. 30% of the patients), this leads to a total group size of 100 patients diagnosed with SIS based on patient history, physical examination, radiographs and MRI arthrography.

Additionally, we expect that around at least 25 patients will be diagnosed with another diagnosis than SIS after MRI-arthrography and radiographs [40,48]. These patients can not be included in the SISTIM study but selected patients will be analyzed separately in distinct research projects, if eligible (trial registry numbers:NTR1545 and NTR2282).

### Outcome measures

The included SIS-patients will be subjected to several diagnostic and experimental tests at the LUMC department of radiology (standard and force task radiographs with EMG, MRI-arthrography) and the Laboratory for Kinematics and Neuromechanics (shoulder kinematics, EMG). The set of measurements is described below and outcome parameters are defined, referring to the mechanisms as summarized in out hypothesis and the primary and secondary study goals (A to J).

#### Basic MRI outcomes (study goals A and J)

For the purpose of assessing eligibility criteria and alternative causes of impingement symptoms, an MRI-arthrography is acquired in each patient. MRI's are reviewed by one of two participating musculoskeletal radiologists at LUMC for inclusion and exclusion criteria and standard clinical evaluation. Additionally, MRI scans will be used to identify potential anatomic/structural causes for SIS symptoms and to assess rotator cuff status (muscle volume, presence of tendinosis/tendinitis, intratendinous, bursal or articular side partial tendon tear, Goutallier score for muscle degeneration) [49,50].

*Main outcome parameters*: inclusion/exclusion of patients, alternative diagnoses leading to SIS symptoms, rotator cuff status, signs of anatomical or structural causes for SIS symptoms.

#### 2D radiographical analyses and EMG (B, C, D, J)

Standard anteroposterior shoulder radiographs enable classification of the acromion shape. Patients' acromion Bigliani classification will be assessed: type I (flat), II (curved) or III (hooked) [14]. We expect an incidence of 30% hooked acromions (type III) in SIS-patients [15].

**Figure 2:**
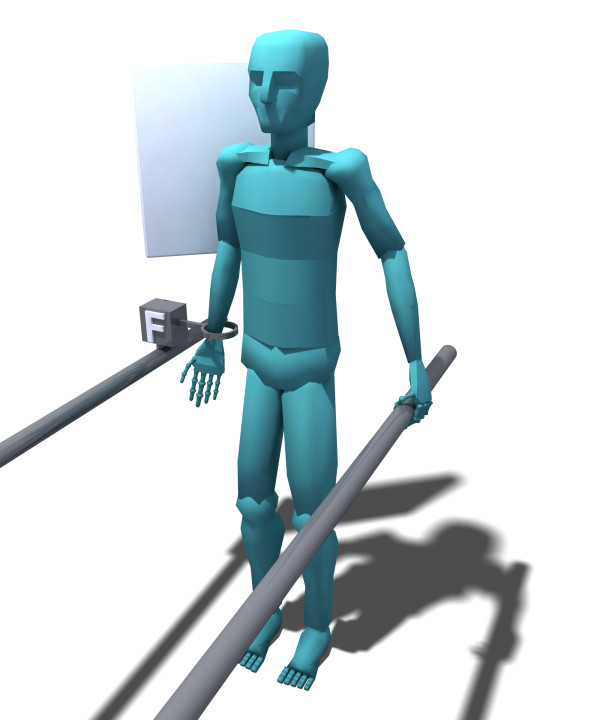
**Set-up for EMG-recorded isometric abduction and adduction force tasks**. Subjects are positioned in front of the radiographic plate, in standing position with the concerning arm in external rotation at his/her side (i.e. hand in frontal plane), enabling the use of this set-up during concomitant acquirement of standard shoulder radiographs. The arm is attached to a 1-dimensional force transducer at the wrist, enabling subject specific force tasks, visual feedback, and equal task force magnitude for abduction and adduction tasks.

Increased subacromial narrowing during arm abduction has been reported in patients with rotator cuff degradation as a consequence of increased Deltoid muscle activation [27,51-54]. In order to observe and study this potential etiological mechanism, radiographs will be acquired in rest position and during EMG-recorded isometric arm abduction and adduction moment tasks of equal force magnitude, using a set-up with a force sensor and visual feedback (Figure 2). We will quantify the subacromial space using the acromiohumeral distance measure (AH), upward migration index (UMI; similar to AH, but corrected for image magnification and patients bony morphological aspects) [55] and spinohumeral centre method (SHC) [56]. Co-activation of medio-caudally directed adductors during active arm abduction has been reported to reduce this humerus cranialisation and consequent pain in rotator cuff patients [5,51,57-60]. Therefore, muscle activation will be controlled for during the three tasks by simultaneous EMG recording with bi-polar surface EMG of the main arm abductor (Deltoids) and adductors (Latissimus Dorsi, Teres Major, Pectoralis Major).

The relative activity of the glenohumeral abductors and adductors will be quantified using the "Activation Ratio" [58-60]. The Activation Ratio (AR_muscle_) of each muscle is determined according to its specific primary function. Muscle activation is either 'in-phase' (A^IP^) or 'out-of-phase' (A^OP^) with respect to its primary moment arm. For example, activation of the medial part of the Deltoid (A_DM_) is defined as 'in phase' during active arm abduction tasks and as 'out-of-phase' during arm adduction tasks. Correspondingly, two average EMG levels are determined for each muscle for 'in phase' and 'out-of-phase' activation with respect to the isometric adduction and abduction moment tasks. Based on these data, subject specific Activation Ratios can be calculated for each muscle (AR_muscle_), Eq 1:

$$AR_{muscle}\;=\;\frac{A_{muscle}^{IP}\;-\;A_{muscle}^{OP}}{A_{muscle}^{IP}\;+\;A_{muscle}^{OP}}\;\left[-\;1\;\leq \;AR_{muscle}\;\leq \;1\right]$$

Consequently, AR's of muscles in healthy subjects are positive and close to 1. In subjects with co-activation of arm adductors during abduction tasks, as has been described for the Latissimus Dorsi and the Teres Major in cuff tear patients, AR's of arm adductor muscles are closer to 0 or even negative. Therefore, we expect to find low adductor Activation Ratios in at least a subgroup of SIS patients, in response to reduced AH.

*Main outcome parameters*: Bigliani acromion classification; Acromiohumeral distance (AH) in rest and during abduction and adduction tasks; muscle-specific EMG Activation Ratios (*AR_deltoid_, AR_lattisimus dorsi_, AR_teres mj_, AR_pectoralis mj_*) for quantification of (adductor) coactivation.

#### 3D radiological analyses (B, F)

Aside from clinical purposes and evaluating inclusion and exclusion criteria, MRI-arthrographies are also acquired to obtain 3D shape parameters of the humerus, scapula and subacromial space with the use of MRI segmentation techniques (Amira 5.3, Visage Imaging Inc., San Diego, CA, USA).

*Main 3D radiological outcome parameters*: 3D shape parameters for humerus, scapula and subacromial space.

#### 3D kinematics and changes in subacromial volume (E, F, G)

Range of Motion (RoM) and 3D motions of forearm, humerus and scapula with respect to the thorax will be recorded by means of an electromagnetic tracking system: 'Flock of Birds' (FoB, Ascension Technology Corp, Burlington, VT, USA) and custom made computer software (FOBVis, Clinical Graphics, Delft, the Netherlands). The FoB obtains 3D kinematical data using sensors on thorax, scapula, humerus, forearm and thorax. After palpatory identifying three dimensional positions of standard bony landmarks of the arm, shoulder and thorax with respect to the sensors for each patient, local bone coordinate systems are created, based on the subject's individual anatomy. The glenohumeral rotation centre is estimated from the position of five scapular bony landmarks using linear regression [61]. The RoM of the following movements is measured: anteflexion, retroflexion, abduction in frontal plane, internal rotation in 0 and 90 degrees of arm abduction and external rotation in 0 and 90° of arm abduction.

3D kinematics and MRI bony segmentation (3D shape parameters of scapula and humerus) will be combined in custom made computer software (Articulus, Clinical Graphics, Delft, the Netherlands) to reconstruct the subacromial space volume and AH during recorded humerus elevations, allowing dynamic measurements of patient-specific subacromial space characteristics [5,62,63]. Additionally, the effect of a subacromial lidocaïne injection on RoM, scapulohumeral rhythm, reconstructed AH and subacromial volume of the affected shoulder will be analyzed.

*Main kinematic outcome parameters*: Passive and active RoM during standardized arm motions (with and without subacromial anaesthetics) of both arms; Scapulohumeral rhythm of affected and healthy arm; Reconstructed changes in AH and subacromial volume during dynamic arm abduction (combining MRI-based shape parameters and 3D RoM measurements).

#### EMG muscle activation patterns (C, D, G)

We will analyze muscle activation patterns as measured by EMG recordings of 10 shoulder muscles, based on Activation Ratio [58,59] and Principal Action parameters [22,51]. Measurements will be performed before and after a subacromial infiltration of lidocaïne (5 ml, 10 mg/ml), to study potential relations between pain during arm abduction and adductor muscle co-activation and arm-scapula kinematics, respectively.

**Figure 3:**
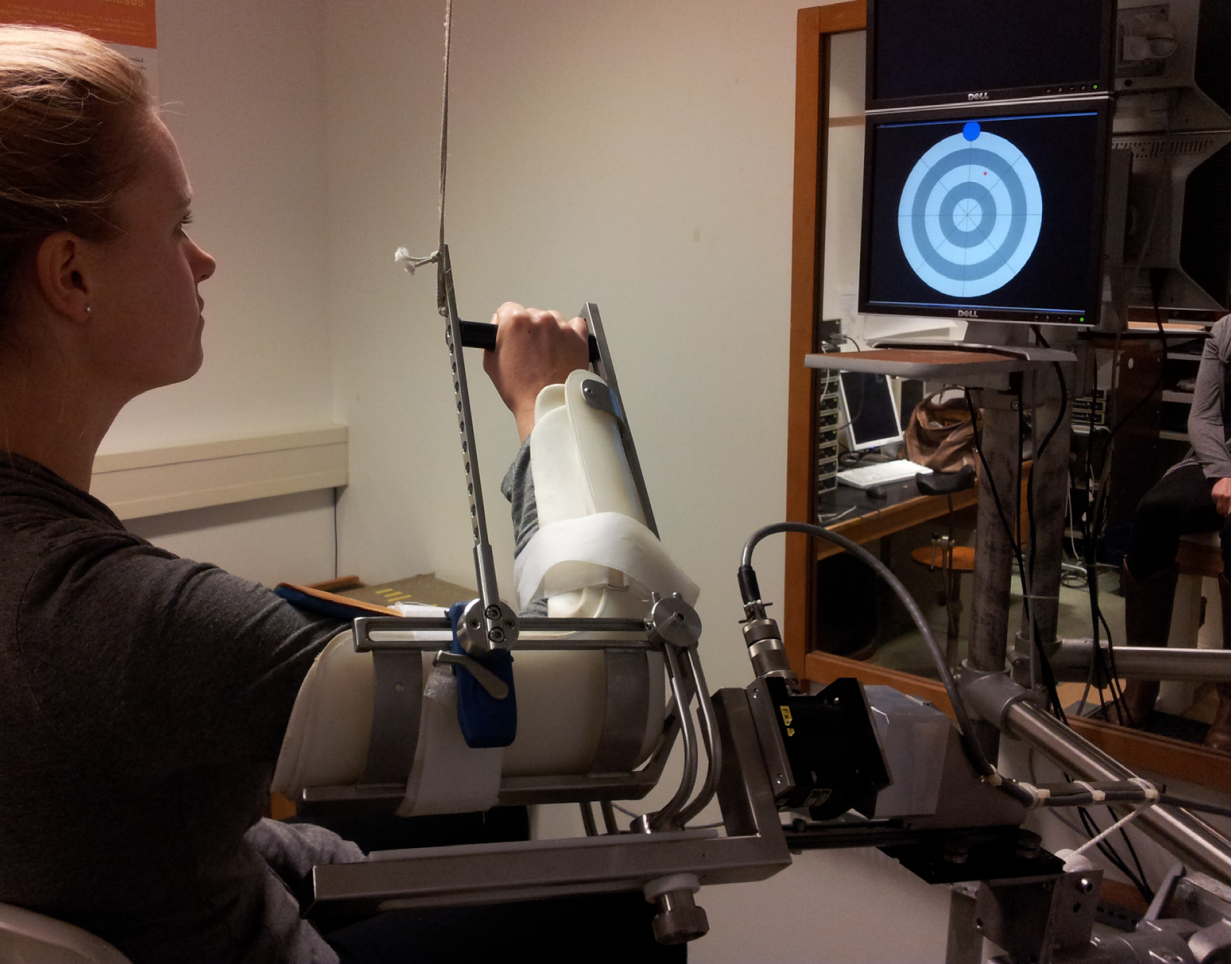
**Experimental setup for isometric arm-shoulder force tasks in 24 directions**. The subject has the arm in a splint, which is connected to a force transducer. Subjects must bring the arm force driven red cursor into the blue target area, which indicates force direction (n = 24 directions) and force magnitude. The exerted force, perpendicular to the humerus long axis, is recorded together with EMG to measure the activity of 10 individual shoulder muscles.

Subjects are seated with the affected arm in a splint with the upper arm in 45° of internal rotation and the elbow in 90° of flexion. The humerus is positioned in 60° of forward elevation and in 30° of external rotation relative to the transverse plane, Figure 3. The splint is attached to a 3D force transducer which is mounted on a sled so that it can move freely in a direction parallel to the humeral longitudinal axis. The arm is fully supported in order to compensate for gravity. Axial rotation of the humerus is mechanically not restricted to prevent the subjects from generating supplementary moments. In this way, patients can only exert forces perpendicular to the longitudinal axis of the humerus.

The subjects are asked to exert a maximal voluntary force (MVF) in 4 equidistant directions with a maximum of 50 N and maintain this force for 2 s using custom made visual feedback software (Matlab, The MathWorks Inc, Natick, MA, USA). The exerted force and force targets are visualized on a display, expressed in cursor that has to be moved to consecutive targets on a wheel in which the spokes denote force directions and the rim denotes the desired force magnitude (Figure 3). Subjects are subsequently asked to exert 75% of the lowest MVF value onto the force transducer for 2 s in each of 24 equidistant directions that are indicated on the display. The same routine of 24 measurements will be performed 30 min after a subacromial injection with lidocaïne. Muscle activations for 10 muscles around the shoulder are recorded in each of the 24 directions during the 2 sets of measurements.

The direction of maximum activity or Principal Action (PA) for each muscle is determined [22,51] and the Activation Ratio of the abductor and adductor muscles similar to the method described above [58,59]. We expect that pain will influence the Principal Action direction of the muscles [51]. Patients with pain will consequently show an increase in activation of the glenohumeral depressors during arm abduction moments (i.e. adductor co-activation as expressed in low Activation Ratio's). The AR's obtained within this 'Principal Action' set-up will be compared to AR's obtained from the derived abduction and adduction tasks as obtained with the EMG set-up applied during the acquirement of radiographs, taking potential experimental dependencies of AR into account.

The second hypothesis is that after lidocaine injection the muscle activation patterns of the patients move toward a normal activation pattern, as expressed in higher adductor Activation Ratio's and near normal Principal Action directions [5,51].

*Main outcome parameters*: Muscle specific Principal Action (PA) parameters and muscle specific Activation Ratios (AR) before and after a subacromial injection with lidocaïne.

#### Model simulation (H)

Impaired cuff function and RoM data obtained from the 3D-Kinematics measurements will be used as input data for the inverse dynamic model simulation with the Delft Shoulder and Elbow Model (DSEM) in order to estimate discrete muscle forces and joint reaction forces with the use of inverse dynamic simulation [64]. Muscle quality and glenohumeral joint stability can be varied and compared to the observations on muscle quality (MRI) and proximal migration (2D radiography) [57].

Similarly to the hypothesized clinical measurements outcomes, we expect to find coactivation of arm adductors on affected shoulders during arm abduction simulations, in combination with altered shoulder muscle force patterns for standardized movements with respect to the control shoulders.

The predicted model muscle forces can be used for validation and interpretation of recorded muscle activations by means of EMG.

#### Patient phenotyping (I)

The radiological and biomechanical outcome measures will be related to patients' clinical status or phenotype. We combine an overall general health outcome measure (i,e, SF36), a regional (e.g. shoulder) outcome measure, and a disease- or condition-specific measure for patient assessment [65].

- SF-36: Questionnaire to measure quality of life, based on physical function, illness, pain and mental health [66].

- Illness Perception Questionnaire (IPQ): measures perception and impact of Illness [67].

- The Disabilities of the Arm, Shoulder and Hand (DASH) score: to quantify impact and functional impairment of shoulder arm and hand function [68].

- Constant Shoulder Score (CS): used by physicians to quantify the severity of symptoms and functional impairment in affected shoulders, compared to the unaffected shoulder [69].

- Western Ontario Rotator Cuff index (WORC): a self-reported outcome measure for assessing shoulder problems as a consequence of rotator cuff disease [70].

- Visual Analogue Scale (VAS) for pain during daily life activities and in rest.

#### Relate outcome measures to pathophysiological mechanisms

**Figure 4:**
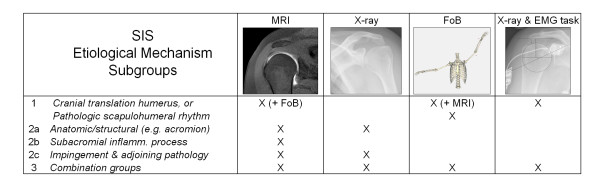
**Schematic outline for relating outcome measures to pathophysiological mechanisms**. We expect to identify one or more of the hypothesized etiological mechanisms in each SIS patient. These mechanisms might be related to the reported variations in SIS symptoms, course, and treatment outcome. (MRI = Magnetic Resonance Imaging, X-ray & EMG task = radiographs during EMG recorded abduction and adduction tasks for measurements of acromiohumeral distance, and FoB = 3D kinematics with Flock of Birds system).

Results of the recorded clinical, radiological and biomechanical measurements will be interpreted and combined in order to classify patients in to the hypothesized etiological subgroups (Figure 4). Ultimately, if a patient has evidence of co-activation of arm depressors with abduction but no signs of shape parameters (e.g. acromion classification) playing a role, this would implicate that an intrinsic and dynamic mechanism is the main pathologic mechanism. On the other hand, evidence of e.g. a type III acromion (hooked) without any signs of relative cranial translation of the humerus would be suggestive of a primarily extrinsic and static/structural cause.

The following scenarios are considered:

1) Dynamically reduced subacromial space, due to (relative) cranial translation of the humerus.

Humerus cranialisation causing encroachment of subacromial tissues will be characterized by limited AH in resting state on standard radiographs, further decrease in AH during abduction tasks, and decreased reconstructed subacromial volume (MRI). In some patients, this cranialisation might be (partially) compensated by I) co-activation of arm adductor muscles, and/or II) altered kinematics of the humerus and the scapula (scapulohumoral rhythm). Nevertheless, instead of a compensation mechanism, altered scapulohumeral rhythm can also be a cause of SIS in some patients (e.g. decreased scapula lateral rotation during arm abduction, with consequent relative cranial translation of the humerus). Pain is suspected to be the main trigger for compensation mechanisms. Therefore, we expect that these compensation mechanisms will be less manifest during the second round of experiments, after a subacromial injection with lidocaine.

Particularly relevant positive outcome measures for this subgroup are: decreased AH, low AR (adductor co-activation), altered Principal Action for adductor muscles, decreased reconstructed subacromial volume during active abduction, degeneration of rotator cuff muscles and altered scapulohumeral rhythm.

2a) Statically reduced subacromial space, due to structural narrowing (classic etiology). The subacromial space can be narrowed as a consequence of structural anatomic variations, e.g. a hooked acromion impinging on the subacromial tissues. These potential causes will be investigated and quantified using shoulder radiographs and (segmented) MRI-arthrographies.

As a consequence of structures impinging on the rotator cuff, compensation mechanisms might be present to prevent further subacromial encroachment, including altered scapulohumeral rhythm (increased lateral rotation or increased posterior tilt during arm abduction) and adductor co-activation during arm abduction. Again, we expect that these compensations mechanisms will be less manifest after a subacromial injection with lidocaine.

Important outcome measures for this subgroup are: shape parameters of scapula (hooked acromion, Bigliani classification, acromial spurs) and humerus, the presence and extend of rotator cuff tendinosis (fibrosis, tendinitis, partial articular or bursal side tear).

2b) Statically reduced subacromial space, as a consequence of subacromial inflammatory processes, without signs of actual structural subacromial narrowing.

In some subjects, symptoms of SIS are related to predominantly intrinsic causes. In these patients, we expect to find little or no anatomic variations impinging on the cuff and no evidently decreased AH. The hypothesized disbalance between subacromial volume and the space needed for subacromial structures can be caused by e.g. subacromial oedema, fibrosis, tendinosis and tendinitis, which will be mainly assessed by means of MRI.

As a consequence of the subacromial inflammatory reaction and pain, patients might have an altered scapulohumeral rhythm and adductor co-activation.

Main outcome measures for characterizing this subgroup are: the presence and extend of rotator cuff tendinosis (fibrosis, tendinitis, partial articular or bursal side tear) and subacromial oedema.

2c) Statically reduced subacromial space due to encroachment of subacromial tissues by an adjoining pathology or other structures than the acromion.

Besides humerus cranialisation and the classical etiologic mechanisms that have been related to SIS, subacromial tissues can be impinged as a consequence of an adjoining pathology or other structures than the acromion. For example, coracoid impingement and subacromial osteophytes in (otherwise asymptomatic) osteoarthritis of the acromioclavicular (AC)-joint have been reported as causes for pain with arm abduction. In our study, these causes will be investigated with the use of radiographs, MRI and 3D-kinematics recordings. Therefore, the most important methods of investigation for this subgroup are: MRI and radiographs to evaluate e.g. AC-osteoarthritis and subacromial osteophytes, impingement on the superior aspect of the glenoid, impingement at the outlet of the shoulder, coracoid impingement, and other (subacromial) pathologies or impinging structures causing a deficiency of subacromial space.

3) Combination groups

As with many diagnoses in general, the cause of SIS symptoms is presumably heterogeneous. We expect that in most patients one of the hypothesized mechanisms will play a main role, but in a subgroup of patients, a combination of 2 or more mechanisms will be causing SIS.

Additionally, we expect to identify specific pathologies other than SIS causing shoulder complaints, including cuff tears, calcifying tendinitis, first stage frozen shoulder, and SLAP lesions. Patients with these pathologies will not be included in the current SISTIM study, but some (cuff tears or calcifying tendinitis) will be analyzed separately in distinct research projects (trial registry numbers: NTR1545 and NTR2282).

#### Statistical analyses

Patient data, including patient characteristics, physical examination, interview, radiological findings, questionnaires, psychological scores, biomechanical measurements and MRI findings will be entered in a database.

With regards to presence of cranial translation of the humerus as detected on radiographs during rest and abduction and adduction tasks, statistical analysis will be performed by a means of repeated measures ANOVA, with the measure of co-contraction controlled as a confounding factor.

For the isometric Principal Action EMG measurements, data are tested by means of a General Linear Model analysis for repeated measures, controlling for factors Muscle, subacromial anaesthetics, sAH and VAS for pain.

RoM in standardized motions will be analyzed with a General Linear Model analysis for repeated measures, controlling for factors VAS pain, subacromial anaesthetic, dAH and sAH. dAH obtained from 3D-kinematics will be analyzed equally.

Additionally, we will use students' unpaired t-tests to compare continuous variables (e.g. patient characteristics, clinical scores, RoM, dAH) between defined pathologic subgroups.

## Discussion

Despite the fact that there is no clear consensus on its etiologic mechanisms nor which combination of diagnostic criteria defines SIS, numerous clinical trials exist on patients with the diagnostic label "SIS". Conflicting in- and exclusion criteria for SIS are used across these heterogeneous studies, complicating interpretation of reported results. Additionally, several pathologies that have a similar patient history, pain pattern and findings on physical examination can be mistakenly diagnosed as SIS [45]. Conclusions of these studies are based on results of patients with varying etiologic mechanisms and for that matter even varying pathologies wrongly diagnosed as SIS, resulting in the wide variety on views with respect to aetiology, diagnosis and treatment of SIS that exists nowadays. Instead of studying the outcomes of various treatment modalities in patients with SIS symptoms, first a detailed analysis of possible underlying pathophysiologic mechanisms is needed. In this way, potential subgroups can be identified, subsequently needing specific approaches in both research and clinical decision-making, with regards to diagnostics and treatment pathways.

The SISTIM study is a cross-sectional descriptive large cohort study in which consecutively included patients will undergo a multitude of biomechanical, kinematical and clinical tests. Patients will be selected using strict eligibility criteria, including radiographs and MRI. As a result a unique set of radiological (radiographs combined with EMG, MRI), biomechanical (muscle activation patterns) and 3D motion data (Flock of Birds) will be available on each individual patient, besides usual clinical data and outcome measures (e.g. CS, WORC). This will give better insight in the etiologic mechanisms in patients with symptoms diagnosed as SIS. Whether there is actual encroachment of subacromial tissues is determined by 1) the volume of these tissues and 2) the available subacromial space (static and dynamic). Both are investigated in our study: the status of subacromial tissues will be investigated with MRI, and we will use bony shape parameters, 3D kinematics and muscle activation patterns, to study their role on the subacromial volume of each patient. As this subacromial space is mainly limited by the scapula and humerus, the interaction of (bony) shape parameters and the (dynamic) position of these structures will be investigated as well.

Our ultimate goal would be to design clinically applicable instruments for differentiating between patients that might benefit from a specific treatment modality (e.g. acromionplasty, depressor training etc.). Therefore, we plan to use our developed experimental methods and classification systems in a subsequent clinical trial, for assessing treatment outcomes of standard care methods in discrete etiological subgroups.

## Competing interests

The authors declare that they have no competing interests.

## Authors' contributions

PdeW, JdG and RN are the principal investigators and designed the SISTIM program in close cooperation. PdeW is also the researcher and holds responsibility for data collection interpretation and publication, and prepared the first draft for this paper.

JdG designed the SISTIM program in close cooperation with RN and PdW and holds primary responsibility for theoretical and experimental part of the study and edited the manuscript.

RN designed the SISTIM program in close cooperation with JdG and PdW and holds primary responsibility for the clinical and administrative part of the study and revised the manuscript critically.

JN, EvA and CV were advisory to the design of the protocol, are responsible for patient recruitment and revised the manuscript critically.

All authors have read and corrected draft versions and approved the final manuscript.

## Pre-publication history

The pre-publication history for this paper can be accessed here:

http://www.biomedcentral.com/1471-2474/12/282/prepub
